# Genetic background to attention deficit and hyperactivity disorder and attention deficit and hyperactivity disorder symptoms at the age of 5 years: the role of sleep duration

**DOI:** 10.1093/sleep/zsad047

**Published:** 2023-03-01

**Authors:** Isabel Morales-Muñoz, E Juulia Paavonen, Katri Kantojärvi, Tommi Härkänen, Outi Saarenpää-Heikkilä, Anneli Kylliäinen, Sari-Leena Himanen, Tiina Paunio

**Affiliations:** Department of Public Health and Welfare, Finnish Institute for Health and Welfare, Helsinki, Finland; Institute for Mental Health, School of Psychology, University of Birmingham, Birmingham, UK; Department of Public Health and Welfare, Finnish Institute for Health and Welfare, Helsinki, Finland; Pediatric Research Center, Child Psychiatry, University of Helsinki and Helsinki University Hospital, Helsinki, Finland; Department of Public Health and Welfare, Finnish Institute for Health and Welfare, Helsinki, Finland; Department of Psychiatry and SleepWell Research Program, Faculty of Medicine, University of Helsinki and Helsinki University Central Hospital, Helsinki, Finland; Department of Public Health and Welfare, Finnish Institute for Health and Welfare, Helsinki, Finland; Pediatric Clinics, Tampere University Hospital, Tampere, Finland; Faculty of Medicine and Life Sciences, Tampere University, Tampere, Finland; Psychology, Faculty of Social Sciences, Tampere University, Tampere, Finland; Department of Clinical Neurophysiology, Tampere University Hospital, Tampere, Finland; Faculty of Medicine and Health Technology, Tampere University, Tampere, Finland; Department of Public Health and Welfare, Finnish Institute for Health and Welfare, Helsinki, Finland; Department of Psychiatry and SleepWell Research Program, Faculty of Medicine, University of Helsinki and Helsinki University Central Hospital, Helsinki, Finland

**Keywords:** PRS for ADHD, inattention, hyperactivity-impulsivity, sleep duration, actigraphy

## Abstract

**Study Objectives:**

We studied the associations between polygenic risk score (PRS) for attention deficit and hyperactivity disorder (ADHD) and (1) ADHD symptoms in 5-year-old children, (2) sleep duration throughout childhood, and (3) the interaction between PRS for ADHD and short sleep duration relative to ADHD symptoms at 5 years.

**Methods:**

This study is based on the population-based CHILD-SLEEP birth cohort (*N* = 1420 children). PRS was used to quantitate the genetic risk for ADHD. Parent-reported ADHD symptoms at 5 years were obtained from 714 children, using the Strengths and Difficulties Questionnaire (SDQ) and the Five-to-Fifteen (FTF). Our primary outcomes were SDQ-hyperactivity and FTF-ADHD total scores. Parent-reported sleep duration was measured at 3, 8, 18, 24 months, and 5 years in the whole sample and actigraphy-based sleep duration at 2 and 24 months in a subsample.

**Results:**

PRS for ADHD associated with SDQ-hyperactivity (*β* = 0.214, *p* = .012) and FTF-ADHD total (*β* = 0.639, *p* = .011), and FTF-inattention and hyperactivity subscale scores (*β* = 0.315, *p* = .017 and *β* = 0.324, *p* = .030), but not with sleep duration at any time point. Significant interactions were found between high PRS for ADHD and parent-reported short sleep throughout childhood in FTF-ADHD total score (*F* = 4.28, *p* = .039) and FTF-inattention subscale (*F* = 4.66, *p* = .031). We did not find any significant interaction between high PRS for ADHD and actigraphy-based short sleep.

**Conclusions:**

Parent-reported short sleep moderates the association between genetic risk of ADHD and ADHD symptoms in early childhood in the general population, so that children with short sleep, in combination with high genetic risk for ADHD, could be at highest risk for ADHD symptoms.

## Graphical abstract

**Graphical Abstract:**
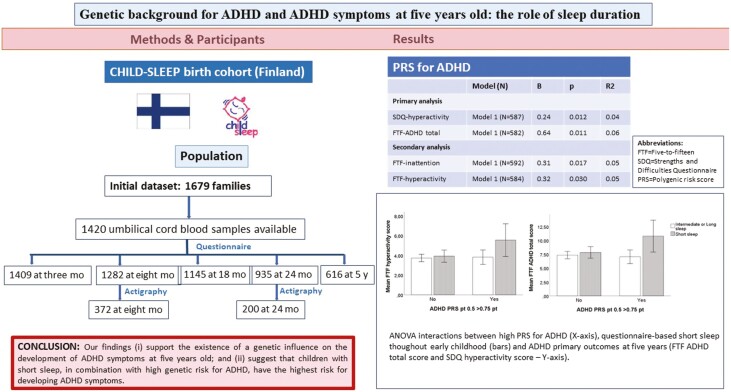

**Statement of Significance**

The polygenic risk score (PRS) for attention deficit and hyperactivity disorder (ADHD) associates with ADHD symptoms. Further, sleep duration could be a causal contributor to ADHD symptoms. However, it remains unclear how PRS for ADHD associates with ADHD symptoms and sleep duration in early childhood, and whether sleep duration contributes to these associations. This is the first study to examine the moderating role of sleep duration in the association between PRS for ADHD and ADHD symptoms at 5 years. Our findings indicate that children with short sleep duration, together with high genetic risk for ADHD, have the highest risk for developing ADHD symptoms at the age of 5 years. Our study highlights the relevance of addressing early sleep duration problems, which could lead to prevention of subsequent ADHD symptoms.

## Introduction

Attention deficit and hyperactivity disorder (ADHD) is a neurodevelopmental disorder that affects around 5% of children and adolescents [1]. Epidemiologic and clinical studies have consistently shown the role of genetic risk factors in the etiology of ADHD. For instance, twin studies indicate that the heritability of ADHD is as high as 70–80% [2]. Common variants seem to comprise a significant fraction of the risk underlying ADHD, as the total contribution of common single nucleotide polymorphisms (SNP) to the heritability of ADHD (i.e. the SNP-based heritability) is approximately 0.10–0.28 [3]. Further, large-scale genome-wide association studies (GWAS) have identified 12 genetic loci associated with risk for ADHD [4]. Based on these studies, one can calculate quantitative polygenic risk scores (PRS) to estimate the individual’s genetic susceptibility for ADHD. A recent systematic review indicates that the odds ratios for PRS associating with ADHD range from 1.22 to 1.76, and the variance explained in dimensional assessments of ADHD traits is 0.7–3.3% [5]. Therefore, this suggests that several other factors also contribute to the risk of ADHD.

Sleep disturbances are a common comorbidity in children with ADHD. Several studies show that individuals with ADHD present with a higher prevalence of sleep problems than other children [6]. More specifically, problems with sleep are reported in about 25–50% of children and adolescents with ADHD [7]. Further, longitudinal studies show that sleep problems in early childhood predict higher risk for ADHD later in childhood [8, 9]. For instance, we recently reported that short sleep duration in early childhood is associated with inattention at 5 years of age, suggesting that inadequate sleep time could be related to ADHD symptoms later on [8]. We also found that parent-reported sleep difficulties at 2 and 5 years, and night awakenings at 5 years were associated with ADHD symptoms at 5 years (i.e. inattention and hyperactivity) [8]. This is line with previous studies showing that shorter sleep time in early childhood is related to ADHD symptoms later on [10, 11]. The relevance of short sleep duration as a risk factor for ADHD is supported by other studies indicating that restricted sleep leads to inattentive symptoms in children and adolescents, and that extending sleep improves attentional and behavioral functioning in adolescents with ADHD [12, 13]. Furthermore, a recent study reported that longer sleep duration (>8.5 h) was associated with a decreased risk of ADHD symptoms in children aged 3–6 years [14]. Therefore, sufficient sleep duration may be an important target in interventions that aim at decreasing symptoms of ADHD. It is also possible that both ADHD symptoms and sleep problems, including short sleep duration, share similar predisposing genetic factors [15].

The PRS for ADHD also associates with depressive symptoms [16], higher body mass index [17], and substance use disorder [18]. However, only one recent study has been performed on sleep traits, which reported that PRS for ADHD was significantly associated with sleep disturbances in children aged 9–10 years [19]. In that study, higher PRS for ADHD in children associated with difficulties in initiating and maintaining sleep and excessive somnolence, but the authors did not report associations with sleep time. In another recent study conducted by Demontis et al. [4], the authors reported a positive genetic correlation between ADHD and insomnia, but this relationship did not appear to generalize to other sleep-related phenotypes, including sleep duration. There are no other previous studies reporting whether genetic risk factors related to ADHD are associated with sleep duration in children. Thus, further research on the contribution of high genetic risk for ADHD to children’s sleep duration and their influence on the development of ADHD symptoms in childhood is required.

Identifying potential interactions between the biological vulnerability to ADHD and deviations in sleep duration in children would contribute to further understanding relevant risk factors for ADHD. To advance the current knowledge of genetic influences on ADHD and sleep in childhood, we designed the current study on a population-based cohort to investigate the associations between PRS for ADHD and (1) ADHD symptoms at 5 years old; (2) sleep duration throughout childhood, (i.e. at 3, 8, 18, 24 months, and 5 years) using both subjective and objective sleep measures; and (3) to investigate the moderating role of sleep duration between the genetic risk for ADHD (i.e. PRS for ADHD) and ADHD symptoms at 5 years of age. We focused on ADHD at 5 years, as this is typically the age when ADHD symptoms become apparent in many children [20]. Although ADHD diagnosis can be considered at the age of 5 years old in some cases, the early detection of symptoms is more crucial compared to early diagnosis. Further, compulsory education in Finland starts at the age of 6 years; thus, 5 years of age is optimal for early detection to prevent the possible additional negative effects associated with schooling (e.g. difficulties in learning and peer relationships). Finally, the delay in early detection could have a negative impact on sleep and ADHD symptomatology. We hypothesized that higher PRS for ADHD would associate with more ADHD symptoms at 5 years and with shorter sleep duration throughout childhood. Furthermore, we hypothesized that short sleep duration in childhood would moderate the association between PRS for ADHD and symptoms of ADHD at 5 years, taking into account the role of sleep disturbances in children, including short sleep duration as a risk factor for the development of ADHD [21].

## Methods

### Participants

This study is based on the CHILD-SLEEP cohort, a population-based birth cohort in Pirkanmaa, southern Finland [22]. For this study, we used parental questionnaires during pregnancy (32nd week), umbilical cord blood samples at birth, questionnaire-based sleep measures at 3, 8, 18 and 24 months, and at 5 years, actigraphy-based sleep measures at 8 and 24 months, and questionnaire-based ADHD-related measures at 5 years. The initial dataset comprised 1679 families, which represented around 34% of the total number of births within the hospital from the targeted area (i.e. 5000 deliveries per year at Tampere University Hospital). From this initial sample, 1438 umbilical cord blood samples were available. Of these, we had questionnaire data from 99.2% (*N* = 1409) infants at 3 months, 90.3% (*N* = 1282) at 8 months, 80.6% (*N* = 1145) at 18 months, 65.8% (*N* = 935) at 24 months, and 43.4% (*N* = 616) at 5 years. Furthermore, there were six children with severe neurological illnesses (Down syndrome *N* = 1, autism *N* = 1, hereditary blindness *N* = 1, mental retardation *N* = 1, severe developmental delay *N* = 2) and four twins that were excluded from the final study, leaving a total of 610 cases with a PRS score and at least one parental questionnaire at 5 years. While all the questionnaires and umbilical blood samples were obtained from the whole sample, actigraphy registrations were conducted in a subsample comprising 372 cases at 8 months, and 200 at 24 months, due to limitations with the resources available to conduct actigraphy recordings in the whole sample. There were no twins in this subsample and cases with neurological illness were excluded (*N* = 3). Concerning parent-reported information on ADHD-related symptoms, this data was available in 714 children at 5 years—and from those, 610 cases also had genetic data.

The CHILD-SLEEP cohort was reviewed and approved by the Pirkanmaa Hospital District Ethics Committee (9/3/2011, Ethical Research Permission Code R11032). All participants (i.e. parents and their child) were included in the study after parents signing the written informed consent in accordance with the Declaration of Helsinki.

### Measures

#### Genetic estimates for ADHD.

DNA samples were extracted from blood leukocytes of cord blood according to standard procedures at the Finnish Institute for Health and Welfare. DNA samples were genotyped with Illumina Infinium PsychArray. Quality control was performed with PLINK 1.9 (www.cog-genomics.org/plink/1.9/). Markers were removed for missingness (>5%) and Hardy-Weinberg equilibrium (*p*-value < 1 × 10^−6^). Individuals were checked for missing genotypes (>5%), relatedness (identical by descent calculation, PI_HAT > 0.2) and population stratification (multidimensional scaling). Principal component analysis (PCA) for population stratification was calculated with PLINK 1.9. Genotyped data was pre-phased with Eagle 2.3.5 [23] and imputed with Beagle 4.1 [24], using the population-specific SISu v2 imputation reference panel.

PRS for ADHD was derived from GWAS where 20 183 ADHD cases and 35 191 controls from iPSYCH and Psychiatric Genomics Consortium (PGC) were studied [4]. The *p*-value threshold for generating the PRS was set at .50 for our analyses, as this was tested to be the best-fit *p*-value threshold for ADHD in previous publications [16, 25]. Further, optimal *p*-value threshold (i.e. *p* < .5) was considered from the model, where PRS explained the highest percentage of the variance (R2) in the outcome. The final variant count of the PRS (pT = .5) was 138 584. We used the PRSice program [26], to estimate PRS for studied individuals.

The PRS score was used in the linear regression models as continuous variable. In the interaction analyses, the PRS for ADHD score was dichotomized at the 75th percentile (i.e. a score of 0.78 points): <75th percentile (low) vs. ≥75th percentile (high), to address those cases who were potentially at the highest genetical risk for ADHD.

#### Subjective sleep measures.

The Brief Infant Sleep Questionnaire (BISQ) was used to evaluate infant sleep quality at each time point [27]. As we were interested in sleep duration, we used both nocturnal sleep hours and daytime sleep hours. They were summed up to calculate total sleep duration per 24 h, which was used as the dependent variable in the subsequent statistical analyses, where we investigated the associations between PRS for ADHD and sleep duration in childhood. Additionally, for subsequent analyses of this study, the average sleep duration throughout childhood was estimated by calculating the mean standard score of total sleep duration by summing the standard scores of the time points at 3, 8, 18 and 24 months, and dividing this by the number of available time points. Then, we dichotomized this mean standard score of total sleep duration, based on the 25th percentile (i.e. a mean *z*-score of −0.46): (>25th percentile representing intermediate or long sleep, vs. ≤ 25th representing short sleep duration), to identify cases with lowest sleep duration throughout early childhood. The parent-reported average sleep times at each time point are reported in Table 1.

**Table 1.** Descriptive data on sleep duration at each time point in short sleepers’ group vs. intermediate or long sleeper group

	Short sleep (*Z*-score <25 pt)				Intermediate or long sleep (*Z*-score ≥25 pt)			
	Mean (*SD*)	Min	Max	*N*	Mean (*SD*)	Min	Max	*N*
Questionnaire-based data								
Total sleep duration at 3 months	13.22 (1.53)	10.00	18.00	158	14.84 (1.53)	10.00	19.00	453
Total sleep duration at 8 months	12.42 (1.03)	9.00	15.00	158	13.65 (1.00)	11.00	20.00	453
Total sleep duration at 18 months	11.70 (0.76)	9.25	13.50	158	12.63 (0.78)	10.50	15.00	453
Total sleep duration at 24 months	11.10 (0.65)	9.00	13.00	158	12.15 (0.74)	10.00	15.00	453
Total sleep duration at 5 years	9.79 (0.62)	8.00	11.25	158	10.55 (0.70)	8.67	13.03	453
Actigraph based data								
Total sleep duration at 8 months	10.70 (0.73)	8.49	12.41	43	12.20 (0.80)	9.90	14.51	152
Total sleep duration at 24 months	10.71 (0.58)	9.34	11.59	43	11.80 (9.35)	9.35	14.54	152

#### Objective sleep measures.

Actiwatch7 was used (CamNtech Ltd, UK). Parents were asked to place the actigraphy on their child’s thigh for 3 consecutive days and to complete the sleep log. Nighttime activity data were scored using the sleep analysis program provided by the manufacturer. Daytime activity data was scored using the nap analysis program. This device uses the Oakley algorithm [28], which has previously been validated in infants [29]. All cases with only one night recorded (*N* = 10) were excluded from the final analyses. Our sleep variable of interest was total actual sleep duration per 24 h, which was calculated as the total sum of nighttime and daytime actual sleep duration. The questionnaire-based and actigraphy-based sleep times correlated significantly (at 8 months: *r* = 0.238, *p* < .001; and at 24 months: *r* = 0.286, *p* < .001). The total score was used for the linear regression models, while for the interaction analyses, the mean standard score for actigraphy total assumed sleep duration in early childhood was calculated by summing the standard scores of each time point available (i.e. eight and 24 months) and dividing this by the number of available time points. Then, we dichotomized the mean standard score for sleep duration based on the 25th percentile (i.e. a *z*-score of −0.52): [≤ 25th percentile (short sleep duration) vs. higher (intermediate or long sleep duration)]. The average sleep times with actigraphy are reported in the Table 1.

#### *ADHD symptoms at 5 years*.

ADHD symptoms were assessed using two parent-reported questionnaires: the Strengths and Difficulties Questionnaire (SDQ) [30], and the Five-to-Fifteen questionnaire (FTF) [31]. The SDQ is a brief behavioral screening questionnaire for children aged between three and 16 and includes 25 questions. Parents rate the statement that best describes their child’s behavior on a three-point scale. We used only the five-item hyperactivity scale, and more specifically the total score. The FTF comprises 181 statements with three response alternatives for those aged between five and 15, related to behavioral or developmental problems. We used 18 items reflecting the same symptoms as found in the DSM-IV criteria for ADHD, comprising the nine-item inattention domain and the nine-item hyperactivity domain. The FTF-inattention total score was the sum of nine inattention items, and the FTF-hyperactivity total score was the sum of nine hyperactivity-impulsivity items. Finally, we also used the FTF-ADHD total score, which is the total sum of the FTF-inattention and hyperactivity total scores.

The primary outcomes for this study were the SDQ-hyperactivity total score and the FTF-ADHD total score at 5 years. Further, as secondary outcomes, we also included FTF-inattention and FTF-hyperactivity subscale scores.

None of the parents reported prior ADHD diagnoses in the children included in this study, and none of the children were using stimulant medication.

#### Covariates.

For the primary regression analyses, we included age (months), sex, and the three genetic top principal components (PC1, PC2, PC3), to adjust the effect for population stratification as covariates. Additionally, we also included the following covariates for our secondary regression analyses: parental education (1 = University/0 = Other), daycare full-time (1 = Yes/0 = No), and screen time per day (in min) as they are all potential confounding factors [32, 33].

### Statistical analyses

Statistical analyses were performed with SPSS V27.0. Socio-demographic variables and a description of the variables of interest appear in Table 2. To deal with missingness, we identified significant factors associated with attrition by conducting logistic regression analyses to predict participation (where 1 indicated participation in the study; 0 indicated non-participation in the study). We used several independent variables as the explanatory factors. We found that the variables associated with participation at 24 months and at 5 years were higher PRS for ADHD score and lower parental education, while participation at 3, 8 and 18 months was associated only with lower parents’ education (Supplementary Table S1). Using the variables associated with dropout, we fitted logistic regression models to determine inverse probability weights (IPW) for each individual and time point using the inverse probability of response (1/logit).

**Table 2.** Socio-demographic factors and variables of interest in children at the age of 5 years old [i.e. with information on FTF or SDQ at 5 years (*N* = 614) or PRS score (*N* = 1420); excluding twins and cases with neurological illness]

	*N* (%^1^)	Mean (*SD*)	Min	Max
PRS_BestFit_	614 (100.0)	−0.072 (0.99)	−3.87	2.87
Gender				
Girls	297 (48.4)	–	–	–
Boys	317 (51.6)	–	–	–
Parental education at 5 years				
University/college degree	294 (47.9)	–	–	–
Other	320 (52.1)	–	–	–
Day care at 5 years				
Full-time	425 (69.2)	–	–	–
Other	189 (30.8)	–	–	–
Screen time at 5 years, mins per day	614 (100.0)	114.84 (48.91)	30.00	351.43
Age, from sample with PRS score (*N* = 1420)				
3 months (months)	1183 (83.3)	3.29 (0.49)	2.40	7.17
8 months (months)	1083 (76.3)	8.25 (0.35)	7.73	13.10
18 months (months)	963 (67.8)	18.67 (1.75)	5.93	23.60
24 months (months)	791 (55.7)	25.06 (1.40)	23.00	33.60
5 years (years)	610 (43.0)	5.71 (0.42)	4.83	5.72
BISQ total sleep hours, from sample with PRS score (*N* = 1420)				
3 months	1098 (77.3)	14.43 (1.68)	9.00	20.50
8 months	1033 (72.7)	13.38 (1.15)	9.00	20.00
18 months	966 (68.0)	12.33 (0.89)	9.25	16.00
24 months	788 (55.5)	11.86 (0.89)	8.25	15.00
5 years	563 (39.6)	10.41 (0.73)	8.00	13.03
ACG total sleep, mins, from sample with PRS score (*N* = 1420)				
8 months	326 (25.5)	11.91 (1.04)	8.49	15.54
24 months	179 (14.0)	11.50 (0.82)	9.34	14.54
SDQ at 5 years				
Hyperactivity total score	587 (96.2)	3.01 (2.33)	0.00	10.00
FTF at 5 years				
ADHD total score	582 (95.4)	7.60 (6.20)	0.00	32.00
Inattention at 5 years	584 (95.7)	3.68 (3.35)	0.00	18.00
Hyperactivity at 5 years	582 (95.4)	3.91 (3.58)	0.00	18.00

PRS_BestFit_, Polygenetic risk score for ADHD, threshold *p* = .5; BISQ, Brief Infant Sleep Questionnaire; ACG, Actigraphy; FTF, Five-to-Fifteen questionnaire; SDQ=, Strengths and Difficulties Questionnaire.

^1^% of those responding at the time point.

To test the first hypothesis that genetic risk for ADHD was related to symptoms of ADHD at 5 years (i.e. two primary outcomes: SDQ-hyperactivity and FTF-ADHD total) and sleep duration throughout childhood, linear regression models were conducted. In these models, the continuous variables representing ADHD symptoms at 5 years and questionnaire-based sleep duration at 3, 8, 18 and 24 months, and 5 years, and actigraphy-based sleep duration at 8 and 24 months were used as dependent variables in separate models. The PRS for ADHD score as a continuous variable was used as the main explanatory variable. In these primary regression analyses, we controlled for age, sex, and PRS three top principal components calculated from genetic data to control for population stratification (Model 1). We report both unweighted and IPW weighted models. Finally, each weighted model was additionally controlled for confounding factors that could be related to sleep or ADHD symptoms. As secondary regression analyses, we added parental education as the covariate in the Model 2, and subsequently screentime and attendance to full-time daycare in the Model 3. The acquired *p* values were adjusted to control false discovery rate (FDR) using the Benjamini–Hochberg procedure for our primary regression analyses, which referred to the investigation of the associations of PRS for ADHD with SDQ-hyperactivity and with FTF-ADHD total at 5 years old (FDR correction for *N* = 2 tests). Further, we conducted additional secondary linear regression analyses with two additional ADHD outcomes at 5 years (i.e. FTF-inattention and FTF-hyperactivity) separately. We did not correct for multiple comparisons in the secondary regression analyses, as these were complementary analyses.

Secondly, to test our hypothesis that short sleep duration in early childhood moderated the associations between PRS for ADHD and ADHD symptoms at 5 years, the interaction terms of the dichotomized PRS for ADHD score and the dichotomized mean standard score for BISQ total sleep duration were added to the ANOVA analyses. This interaction was tested for each of the four dependent variables (i.e. two primary and two secondary ADHD outcomes at 5 years). Finally, similar analyses were conducted using the dichotomized mean standard score for actigraphy total actual sleep duration in the interaction terms in the ANOVA analyses.

Finally, we also studied whether there were any significant interaction effects between the PRS for ADHD score and sex, as sex was found to be a significant covariate in all the models related to ADHD symptoms.

## Results

### PRS for ADHD and ADHD symptoms at 5 years

In relation to our primary outcomes, the weighted linear regression analyses showed that PRS for ADHD was significantly associated with both ADHD measures at 5 years old in Model 1 when we controlled for PC1, PC2 and PC3, age and sex (i.e. SDQ-hyperactivity: *β* = 0.241; *p* = .012; and the FTF-ADHD total score: *β* = 0.639 *p* = .011). After Benjamini–Hochberg multiple testing correction, these associations remained significant (FDR < 0.05).

We also tested the weighted linear regressions for the secondary outcomes and found that PRS for ADHD was also significantly associated with both secondary ADHD measures in Model 1 (i.e. FTF-inattention: *β* = 0.315, *p* = .017; and FTF-hyperactivity: *β* = 0.324, *p* = .030). See Table 3 for all statistical associations with primary and secondary outcome variables.

**Table 3.** Weighted linear regression analyses to test the association between PRS for ADHD and parent-reported ADHD symptoms at five years excluding twins (*N* = 4) and cases with neurological illness (*N* = 6)

	PRS ADHD, *P* = .5			
	Valid *N*	*B* (*SE*)	*P*-value	*R* ^2^
Primary analysis				
SDQ-hyperactivity	587	0.241 (0.096)	.012^*^	0.041
FTF-ADHD total	582	0.639 (0.249)	.011^*^	0.063
Secondary analysis				
FTF-inattention	592	0.315 (0.131)	.017^*^	0.054
FTF-hyperactivity	584	0.324 (0.149)	.030^*^	0.053

Final sample *N* = 610. Model 1: controlled for PC1, PC2, and PC3, age and sex.

*β*, unstandardized beta; *SE*, Standard error.

^*^After FDR correction, the associations between PRS for ADHD and the primary outcomes (i.e. SDQ-Hyperactivity and FTF-ADHD total score) remained significant (FDR < 0.05).

Sex was significantly related to ADHD symptoms at the age of 5 years in all the studied models (all *p*’s < .001), but there were no significant interactions between PRS for ADHD and sex regarding ADHD symptoms scores (all *p*’s > .180).

Finally, we calculated the weighted linear regression models by additionally controlling for parental education (i.e. Model 2), and daycare and screen time at 5 years (i.e. Model 3). Briefly, all the significant associations remained similar. The statistical parameters for Model 2 and 3 are reported in Supplementary Table S2.

All the unweighted linear regression models for both primary and secondary outcomes are presented in Supplementary material (Supplementary Table S3). Briefly, the results were similar to those gathered in the weighted regression models.

### PRS for ADHD and parent-reported and actigraphy-based sleep duration throughout childhood

The linear regression analyses did not show any significant associations between PRS for ADHD and parent-reported sleep duration (see Table 4 for Model 1; Supplementary Table S4 for Model 2; and Supplementary Table S5 for unweighted analyses). Moreover, there were no significant associations between PRS for ADHD and actigraphy-based total sleep duration in the linear regression models (see Supplementary Table S6 for unweighted analyses). Sex was not significantly related to sleep time in any of the time points, and there were no significant interactions between sex and PRS for the ADHD score.

**Table 4.** Weighted linear regression analyses between PRS for ADHD and questionnaire-based total sleep duration at 3, 8, 18, and 24 months and at 5 years (Model 1), excluding twins (*N* = 4) and cases with neurological illness (*N* = 6)

	PRS for ADHD, *P* = .5		
	*β* (*SE*)	*P*-value	*R* ^2^
Total sleep 3 months	0.003 (0.051)	.958	0.008
Total sleep 8 months	0.040 (0.037)	.272	0.009
Total sleep 18 months	−0.005 (0.029)	.859	0.007
Total sleep 24 months	0.004 (0.031)	.907	0.007
Total sleep 5 years	−0.041 (0.033)	.217	0.074

Model 1: controlled for PC1, PC2, and PC3, age and sex. Valid *N* = 1096 at 3 months, *N* = 1031 at 8 months, *N* = 958 at 18 months, *N* = 787 at 24 months, *N* = 563 at 5 years.

*β*, unstandardized beta; *SE*, Standard error.

### Sleep duration as a moderator in the associations between PRS for ADHD and ADHD symptoms

**Figure 1.**
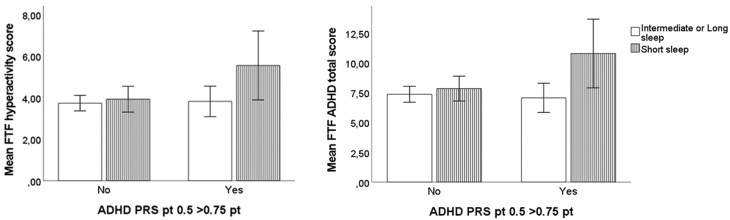
ANOVA interactions between high PRS for ADHD, questionnaire-based short sleep duration throughout early childhood and ADHD primary outcomes (i.e. SDQ-hyperactivity and FTF-ADHD total score) at 5 years. (A) Displays the differences in relation to SDQ-hyperactivity score and shows that those children with both short sleep duration and high PRS for ADHD present the highest scores in SDQ-hyperactivity at the age of five. (B) Displays the differences in relation to the FTF-ADHD total score and shows that those children with both short sleep duration and high PRS for ADHD present the highest scores in FTF-ADHD total score at the age of five. The confidence interval represents ± 2 SE. *X*-axis refers to the PRS for ADHD score (*p*-value threshold .50), which was dichotomized at the 75th percentile: ≥75th percentile (i.e. high = Yes) vs. lower (= No). The *Y*-axis refers to the total mean score for the outcome at the age of five.

In the unweighted/weighted ANOVA analyses, short sleep duration in early childhood significantly moderated the association between high PRS for ADHD and ADHD symptoms, so that the highest FTF-ADHD total scores were found in cases with highest genetic risk for ADHD and short sleep duration throughout childhood (unweighted model: *F* = 6.32, *p* = .012, and weighted model: *F* = 4.28, *p* = .039) (see Figure 1B). We also found a significant interaction between the highest PRS score for ADHD and short sleep duration throughout childhood relative to FTF-inattention at 5 years (unweighted model: *F* = 5.84, *p* = .016, weighted model: *F* = 4.66, *p* = .031), and between FTF-hyperactivity at 5 years (unweighted model *F* = 4.346, *p* = .038, weighted model: *F* = 2.87, *p* = .091) (see Supplementary Figure S1A and B). The interaction between highest genetic risk for ADHD and short sleep duration throughout childhood relative to SDQ-hyperactivity was not significant (*F* = 1.67, *p* = .200, and weighted model *F* = 1.392, *p* = .238), although there was a similar trend.

When we investigated actigraphy-based short total sleep duration as a potential moderator, we did not find any significant associations with the 25th percentile as the cut-off for short sleep duration (see Supplementary Table S7).

## Discussion

Our main findings indicated that genetic liability to ADHD associated with ADHD symptoms at 5 years, but not with sleep duration throughout early childhood, in this population-based birth cohort from Finland. There was, however, a significant interaction between short sleep duration throughout childhood with the PRS for ADHD in relation to ADHD symptoms. This is the first study to report that short sleep duration in early childhood moderates the association between genetic liability to ADHD and ADHD symptoms at 5 years, so that persistent short sleep duration increases the risk attributed to the genetic risk relative to ADHD.

First, and following our hypothesis, we found that the genetic liability to ADHD was associated with ADHD symptoms at 5 years, including hyperactivity and inattention. This is supported by previous research where PRS for ADHD has shown positive associations with both hyperactive-impulsivity and inattention in children at aged seven and ten in the general population [34]. A recent systematic review on the topic identified 44 relevant studies that supported the accumulating evidence that PRS for ADHD associates with ADHD diagnosis and ADHD symptoms in children, adolescents, and adults, in addition to other phenotypes [5]. Therefore, this study and previous studies demonstrate that the PRS for ADHD is related to elevated ADHD symptoms. To note, most of the research investigating the links between PRS for ADHD and ADHD has been focused on school-aged children, probably due to the fact that the prevalence of ADHD in very young children (aged < 6 years) is less well studied [35]. Here, we contribute to the existing literature with new evidence indicating that PRS for ADHD is also associated with ADHD symptoms at the age of five.

Second, and contrary to our hypothesis, the genetic liability to ADHD did not associate with sleep duration at any time point during childhood. Our hypothesis was formulated based on previous existing research, including our own [8], indicating that short sleep duration is associated with ADHD symptoms in children, in addition to recent evidence reporting that higher PRS for ADHD in children associates with symptoms of insomnia [19]. However, ours is the first study to investigate the associations between PRS for ADHD and sleep duration in childhood. Based on our results, we can conclude that PRS of ADHD is not robustly associated with sleep duration throughout childhood, suggesting that the influence of genetic risk for ADHD on sleep duration could be relatively low in children, compared to the contribution of environmental factors.

In fact, there is strong support for the role of environmental factors in the development of ADHD symptoms, including perinatal family and parental factors [33], substance exposure during pregnancy and low birth weight [36], or family adversities [37]. Therefore, the associations between sleep duration in childhood and ADHD from epidemiological and clinical studies are likely to be more strongly related to shared environmental factors and to the effect of insufficient sleep on brain functions, rather than to genetic factors. Similarly, in a recent study by us on the links between PRS for chronotype and sleep duration throughout childhood [38], we found that PRS for chronotype associated with sleep-onset latency and bedtime, but not with sleep duration. Consistently with that data, previous findings from 18-month-old twins support the idea that short sleep duration may be mainly explained by common shared environmental factors [39].

Third, sleep duration throughout childhood moderated the associations between PRS for ADHD and ADHD symptoms at 5 years old. Therefore, our results suggest that those children presenting with short sleep duration throughout childhood, in combination with high genetic liability to ADHD, have the highest risk for developing ADHD symptoms at 5 years. One of the potential explanations for why short sleep duration might constitute a moderator in the link between PRS for ADHD and subsequent ADHD symptoms can be found in the shared underlying mechanisms for ADHD and sleep disturbance [40]. For instance, a recent study reported that smaller volumes in the cognitive control system and the salience/ventral attention system constitutes a common neural correlate linking both ADHD and sleep disturbance [41]. Subsequently, impairment at these cortical levels would lead to increased ADHD symptoms later in life. However, it is for further studies to acquire increased understanding of the common underlying mechanisms between sleep and ADHD, and especially in relation to the genetic liability to ADHD.

This study has a number of strengths, including the longitudinal study setting from a population-based sample, the use of several measurement points that cover sleep through childhood, and the characterization of sleep duration using both parent-reported and actigraphy-based measures. Moreover, none of the study participants were prescribed ADHD medication, so it does not confound the findings reported in this study. However, there are also some limitations. First, we used parent-reported information on children’s ADHD symptoms at 5 years, but additional information from daycare could be valuable. Second, there are other confounding factors that might influence our results, such as parental (e.g. parenting styles, parental mental health) or children’s developmental factors (e.g. temperament). Third, the sample of children with actigraphy recording was considerably smaller than the complete sample size. Further, there might be some biases associated with the participation in the actigraphy sample, which might explain why we did not find any significant interaction with actigraphy-based short sleep, as opposed to questionnaire-based short sleep. For this study, we used the 25th percentile, which in the case of our actigraphy data, and probably due to the small sample size, did not discriminate short sleepers from average sleepers well enough, considering that a small deviance from sleep need does not usually affect daytime functioning. In future studies, it is important to study which specific cut-off for short sleep duration in early childhood has the best predictive validity in terms of negative daytime consequences. Thus, our group will further investigate which are the most accurate cut-offs. Therefore, the negative findings with actigraphy data in this study should be cautiously interpreted. Fourth, in this study we solely focused on sleep duration as a potential target in the associations between PRS for ADHD and ADHD symptoms; however, future studies should also investigate the role of other relevant sleep disturbances. Fifth, in this study we were only able to assess common genetic variation, but rare genetic variants may also play a role, and therefore this should also be taken into consideration for future research. Sixth, the *R*^2^ values when all significant covariates were included in the model were low (i.e. <0.10), suggesting that the independent variable (i.e. PRS for ADHD) does not explain much of the variability in the dependent variable. However, *R*^2^ values are usually low in human behavior studies. It is likely that several factors that were not studied in this study (e.g. environmental, developmental, or parental factors, among others) explain some of the variability in the dependent variable. However, the aim of the study was to explore the association between PRS for ADHD and ADHD symptoms and whether sleep duration moderated it and therefore the models that we report here are valid for the selected purpose. Finally, in this study we focused only on sleep duration moderating the association between PRS for ADHD and ADHD symptoms. However, and considering that sleep duration is also related to other sleep parameters, such as bedtime, sleep fragmentation or sleep-onset latency, future studies should investigate the potential moderating role of a wider range of sleep aspects.

In summary, we found that PRS for ADHD associated with ADHD symptoms, including hyperactivity and inattention at 5 years, but not with sleep duration throughout childhood. Furthermore, we observed that the associations between PRS for ADHD and ADHD symptoms at 5 years were moderated by short sleep duration throughout early childhood. Our findings support the argument for the existence of a genetic influence on the development of ADHD symptoms at 5 years old. Further, our findings suggest that children with short sleep duration, in combination with high genetic risk for ADHD, have the highest risk for developing ADHD symptoms. Therefore, our findings potentially highlight the relevance of addressing sleep duration problems in early childhood and studying the implementation of early sleep interventions that could lead to improvement and/or prevention of subsequent ADHD symptoms.

## Supplementary Material

zsad047_suppl_Supplementary_Material

## Data Availability

Due to Finnish federal legislation on personal data protection in medicalresearch, the original research data cannot be made available online, butdata can potentially be shared with Material Transfer Agreement. Requestsand collaboration initiatives can be directed to the Board of The Child Sleep BirthCohort Study. Please contact Prof Tiina Paunio (tiina.paunio@helsinki.fi).
