# Supplementary material for: Genetic background to attention deficit and hyperactivity disorder and attention deficit and hyperactivity disorder symptoms at the age of 5 years: the role of sleep duration
Source: Sleep. 2023 Mar 1;46(7):zsad047. doi: 10.1093/sleep/zsad047 (PMC10799321; doi:10.1093/sleep/zsad047)
Supplement: zsad047_suppl_Supplementary_Material [file zsad047_suppl_supplementary_material.docx]

**Genetic background to ADHD and ADHD symptoms at the age of five years: the role of sleep duration**

Isabel Morales-Muñoz,^1,2^* E Juulia Paavonen,^1,3^* Katri Kantojärvi,^1,4^ Tommi Härkänen,^1^ Outi Saarenpää-Heikkilä,^5,6^ Anneli Kylliäinen,^7^ Sari-Leena Himanen,^8,9^ Tiina Paunio^1,4^

*^1^The Department of Public Health and Welfare, Finnish Institute for Health and Welfare, Helsinki, Finland.*

*^2^Institute for Mental Health, School of Psychology, University of Birmingham, United Kingdom.*

*^3^Pediatric Research Center, Child Psychiatry, University of Helsinki and Helsinki University Hospital, Finland.*

*^4^Department of Psychiatry and SleepWell Research Program, Faculty of Medicine, University of Helsinki and Helsinki University Central Hospital, Finland.*

*^5^Pediatric Clinics, Tampere University Hospital, Finland.*

*^6^Faculty of Medicine and Life Sciences, Tampere University, Tampere, Finland.*

*^7^Psychology, Faculty of Social Sciences, Tampere University, Finland.*

*^8^Department of Clinical Neurophysiology, Tampere University Hospital, Finland.*

*^9^Faculty of Medicine and Health Technology, Tampere University, Tampere, Finland.*

**These authors contributed equally to this paper*

Address correspondence to:

Isabel Morales-Muñoz PhD, Institute for Mental Health, School of Psychology, University of Birmingham, 52 Pritchatts Road, B15 2SA, Birmingham, United Kingdom; Email: I.Morales-Munoz@bham.ac.uk.

Tiina Paunio, Professor, Department of Health, National Institute for Health and Welfare, and Department of Psychiatry and SleepWell Research Program, Faculty of Medicine, University of Helsinki and Helsinki University Hospital, Finland. Email: [tiina.paunio@thl.fi](mailto:tiina.paunio@thl.fi) and [tiina.paunio@helsinki.fi](mailto:tiina.paunio@helsinki.fi)

**Table S1.** Differences in PRS for ADHD and socio-demographic variables between non-participating and participating subjects in the study.

|  |  |  | **Non-participating**  **group** | **Participating group** | ***Non-participating vs. participating*** |  |
| --- | --- | --- | --- | --- | --- | --- |
| ***At 5 years*** | | | | | | |
|  |  | ***N*** | ***Mean (SD)*** | ***Mean (SD)*** | ***OR (95% CI)*** | ***p*** |
| PRS for ADHD |  | 818/620 | 0.05 (1.01) | -0.07 (0.99) | 0.88 (0.79 – 0.98) | 0.017 |
| Gestational age |  | 754/683 | 40.07 (1.18) | 40.05 (1.14) | 0.98 (0.90 – 1.08) | 0.722 |
|  |  |  | ***N(%)*** | ***N (%)*** | ***OR (95% CI)*** | ***p*** |
| Sex | Male |  | 417 (52.9%) | 375 (52.5%) | 1.01 (0.82 – 1.23) | 0.946 |
|  | Female |  | 372 (47.1%) | 339 (47.5%) |  |  |
| Parental education | University |  | 392 (40.8%) | 339 (47.6%) | 0.76 (0.62 – 0.92) | 0.005 |
|  | Other |  | 569 (59.2%) | 373 (52.4%) |  |  |
| ***At 3 months*** | | | | | | |
|  |  |  | ***Mean (SD)*** | ***Mean (SD)*** | ***OR (95% CI)*** | ***p*** |
| PRS for ADHD |  | 197/1241 | -0.02 (0.99) | 0.00 (1.00) | 1.02 (0.88 – 1.19) | 0.786 |
| Gestational age |  | 68/1369 | 40.08 (1.10) | 40.06 (1.17) | 0.98 (0.80 – 1.21) | 0.879 |
|  |  |  | ***N (%)*** | ***N(%)*** | ***OR (95% CI)*** | ***p*** |
| Sex | Male |  | 42 (55.3%) | 750 (52.6%) | 1.19 (0.74 – 1.92) | 0.467 |
|  | Female |  | 34 (44.7%) | 677 (47.4%) |  |  |
| Parental education | University |  | 89 (35.6%) | 642 (45.1%) | 0.67 (0.51 – 0.89) | 0.005 |
|  | Other |  | 161 (64.4%) | 781 (54.9%) |  |  |
| ***At 8 months*** | | | | | | |
|  |  |  | ***Mean (SD)*** | ***Mean (SD)*** | ***OR (95% CI)*** | ***p*** |
| PRS for ADHD |  | 306/1132 | 0.07 (0.96) | -0.02 (1.01) | 0.92 (0.81 – 1.04) | 0.200 |
| Gestational age |  | 187/1250 | 39.98 (1.18) | 40.07 (1.16) | 1.07 (0.93 – 1.22) | 0.336 |
|  |  |  | ***N (%)*** | ***N (%)*** | ***OR (95% CI)*** | ***p*** |
| Sex | Male |  | 111(55.0%) | 681 (52.3%) | 1.14 (0.84 – 1.54) | 0.399 |
|  | Female |  | 91 (45.0%) | 620 (47.7%) |  |  |
| Parental education | University |  | 138 (37.0%) | 593 (45.6%) | 0.70 (0.55 – 0.89) | 0.003 |
|  | Other |  | 235 (63.0%) | 707 (55.0%) |  |  |
| ***At 18 months*** | | | | | | |
|  |  |  | ***Mean (SD)*** | ***Mean (SD)*** | ***OR (95% CI)*** | ***p*** |
| PRD for ADHD |  | 425/1103 | 0.04 (1.00) | -0.02 (1.00) | 0.95 (0.84 – 1.06) | 0.335 |
| Gestational age |  | 318/1119 | 40.03 (1.14) | 40.07 (1.17) | 1.03 (0.93 – 1.15) | 0.554 |
|  |  |  | ***N (%)*** | ***N (%)*** | ***OR (95% CI)*** | ***p*** |
| Sex | Male |  | 182 (53.5%) | 610 (52.5%) | 1.06 (0.83 – 1.35) | 0.646 |
|  | Female |  | 158(47.5%) | 553 (47.5%) |  |  |
| Parental education | University |  | 186 (36.3%) | 545 (46.9%) | 0.65 (0.52 – 0.80) | <0.001 |
|  | Other |  | 326 (63.7%) | 616 (53.1%) |  |  |
| ***At 24 months*** | | | | | | |
|  |  |  | ***Mean (SD)*** | ***Mean (SD)*** | ***OR (95% CI)*** | ***p*** |
| PRS for ADHD |  | 613/825 | 0.08 (0.99) | -0.06 (1.01) | 0.87 (0.78 – 0.97) | 0.010 |
| Gestational age |  | 624/913 | 40.05 (1.17) | 40.07 (1.16) | 1.01 (0.92 – 1.11) | 0.778 |
|  |  |  | ***N (%)*** | ***N (%)*** | ***OR (95% CI)*** | ***p*** |
| Sex | Male |  | 291 (52.6%) | 501 (52.7%) | 1.00 (0.81 – 1.24) | 0.974 |
|  | Female |  | 262 (47.4%) | 449 (47.3%) |  |  |
| Parental education | University |  | 262 (36.2%) | 469 (49.4%) | 0.58 (0.48 – 0.71) | <0.001 |
|  | Other |  | 462 (63.8%) | 480 (50.6%) |  |  |

**Table S2.** Weighted associations between PRS for ADHD and parent-reported ADHD symptoms at five years, for Models 2 and 3, excluding twins (N=4) and cases with neurological illness (N=6).

|  | |  | |  | PRS ADHD, p=0.5 | | |  |
| --- | --- | --- | --- | --- | --- | --- | --- | --- |
|  |  | N | | N | β (SE) | p-value | | R^2^ |
| **Primary analysis** | |  | |  |  |  | |  |
| SDQ-hyperactivity | | Model 2 | | 587 | 0.212 (0.095)^1^ | 0.027 | | 0.056 |
|  | | Model 3 | | 587 | 0.188 (0.092)^2^ | 0.042 | | 0.095 |
| FTF-ADHD total | | Model 2 | | 582 | 0.585 (0.247)^1^ | 0.018 | | 0.078 |
|  |  | Model 3 | | 582 | 0.525 (0.242)^2^ | 0.031 | | 0.106 |
| **Secondary analysis** | | | |  |  |  | |  |
| FTF-inattention | | Model 2 | | 582 | 0.285 (0.130)^1^ | 0.029 | | 0.068 |
|  | | Model 3 | | 582 | 0.250 (0.127)^2^ | 0.050 | | 0.106 |
| FTF-hyperactivity | | | Model 2 | 584 | 0.298 (0.148)^1^ | 0.045 | 0.062 | |
|  | | | Model 3 | 584 | 0.274 (0.147)^2^ | 0.063 | 0.074 | |

^1^ Model 2: controlled for PC1, PC2 and PC3, age, sex, parental education

^2^ Model 3: controlled for PC1, PC2 and PC3, age, sex, parental education, day care and screen time

β=Unstandardized beta; SE=Standard error

Models 1-3 Valid sample size for SDQ N=587, for FTF N=582-584

**Table S3.** Unweighted associations between PRS for ADHD (PRS ADHD, p=0.5) and parent-reported ADHD symptoms at 5 years, excluding twins (N=4) and cases with neurological illness (N=6).

| **Primary analysis** | | | |
| --- | --- | --- | --- |
|  | B (SE) | p-value | R^2^ |
| SDQ-hyperactivity | 0.240 (0.095) | 0.009 | 0.041 |
|  | 0.227 (0.096) | 0.019 | 0.056 |
|  | 0.202 (0.095) | 0.034 | 0.096 |
| FTF-ADHD total | 0.681 (0.254) | 0.008 | 0.063 |
|  | 0.616 (0.253) | 0.015 | 0.078 |
|  | 0.562 (0.251) | 0.026 | 0.106 |
| **Secondary analysis** | | | |
| FTF-inattention | 0.339 (0.138) | 0.014 | 0.053 |
|  | 0.304 (0.138) | 0.028 | 0.068 |
|  | 0.272 (0.136) | 0.080 | 0.105 |
| FTF-hyperactivity | 0.341 (0.147) | 0.021 | 0.044 |
|  | 0.311 (0.147) | 0.035 | 0.063 |
|  | 0.289 (0.147) | 0.050 | 0.075 |

Model 1: controlled for PC1, PC2 and PC3, age and sex

Model 2: controlled for PC1, PC2 and PC3, age, sex, parental education

Model 3: controlled for PC1, PC2 and PC3, age, sex, parental education, day care and screen time

B=Unstandardized beta; SE=Standard error

Models 1-3 Valid sample size for SDQ N=587, for FTF N=582 (FTF-inattention and FTF-ADHD total)-584 (FTF-hyperactivity)

**Table S4.** Weighted associations between PRS ADHD and questionnaire-based total sleep duration at three, eight, 18, and 24 months and at five years for Model 2, excluding twins (N=4-10 depending on the time point) and cases with neurological illness (N=6).

|  |  | | PRS ADHD, p=0.5 | |
| --- | --- | --- | --- | --- |
|  | β (SE) | p-value | | R^2^ |
| Total sleep 3 months | -0.002 (0.051) | 0.965 | | 0.007 |
| Total sleep 8 months | 0.039 (0.037) | 0.285 | | 0.009 |
| Total sleep 18 months | -0.004 (0.029) | 0.884 | | 0.007 |
| Total sleep 24 months | 0.001 (0.031) | 0.985 | | 0.008 |
| Total sleep 5 years | -0.032 (0.122) | 0.793 | | 0.017 |

Model 2: controlled for PC1, PC2 and PC3, age, sex, parental education

β=Unstandardized beta; SE=Standard error

Valid N=1096 at 3 mo, N=1031 at 8 mo, N=958 at 18 mo, N=787 at 24 mo, N=563 at 5 years

**Table S5.** Unweighted associations between PRS ADHD and questionnaire-based total sleep duration at three, eight, 18, and 24 months and at five years, excluding twins (N=4-10 depending on the time point) and cases with neurological illness (N=6).

|  |  | PRS ADHD, p=0.5 | | |
| --- | --- | --- | --- | --- |
|  | β (SE | | p-value | R^2^ |
| Total sleep 3 months | 0.000 (0.051)^1^ | | 0.998 | 0.009 |
|  | -0.001 (0.051)^2^ | | 0.989 | 0.009 |
| Total sleep 8 months | 0.044 (0.036)^1^ | | 0.226 | 0.010 |
|  | 0.042 (0.036)^2^ | | 0.242 | 0.010 |
| Total sleep 18 months | -0.002 (0.029)^1^ | | 0.942 | 0.007 |
|  | -0.002 (0.029)^2^ | | 0.957 | 0.007 |
| Total sleep 24 months | 0.007 (0.032)^1^ | | 0.820 | 0.007 |
|  | 0.004 (0.032)^2^ | | 0.905 | 0.008 |
| Total sleep 5 years | -0.041 (0.030)^1^ | | 0.174 | 0.070 |
|  | -0.041 (0.030)^2^ | | 0.171 | 0.070 |

^1^ Model 1: controlled for PC1, PC2 and PC3, age and sex

^2^ Model 2: controlled for PC1, PC2 and PC3, age, sex, parental education

Valid N=1096 at 3 mo, N=1031 at 8 mo, N=958 at 18 mo, N=787 at 24 mo, N=563 at 5 years

β=Unstandardized beta; SE=Standard error

**Table S6.** Unweighted associations between PRS for ADHD and actigraphy-based total actual sleep duration at 8 and 24 months, excluding cases with neurological illness (N=3).

|  |  | PRS ADHD, p=0.5 | | |
| --- | --- | --- | --- | --- |
|  | β (SE) | | p-value | R^2^ |
| Total sleep 8 months | 0.073 (0.059)^1^ | | 0.213 | 0.007 |
|  | 0.073 (0.059)^2^ | | 0.217 | 0.007 |
| Total sleep 24 months | -0.085 (0.068)^1^ | | 0.211 | 0.048 |
|  | -0.092 (0.068)^2^ | | 0.181 | 0.054 |

^1^ Model 1: controlled for PC1, PC2 and PC3, age and sex,

^2^ Model 2: controlled for PC1, PC2 and PC3, age, sex, parental education

Valid N=333 at 8 mo, N=143 at 24 mo

β=Unstandardized beta; SE=Standard error

**Table S7.** ANOVA interactions between high PRS for ADHD and short actigraphy-based total assumed sleep (25^th^ percentile) in relation to ADHD outcomes at 5 years, excluding cases with neurological illness (N=3).

| **PRS ADHD X actigaph-based short sleep 25^th^ pc** | | |
| --- | --- | --- |
| **Primary outcomes** | | |
|  | F | p-value |
| SDQ-Hyperactivity | 0.000 | 0.983 |
| FTF-ADHD total | 0.474 | 0.492 |
| **Secondary outcomes** | | |
| FTF-inattention | 0.374 | 0.542 |
| FTF-impulsivity | 0.353 | 0.553 |


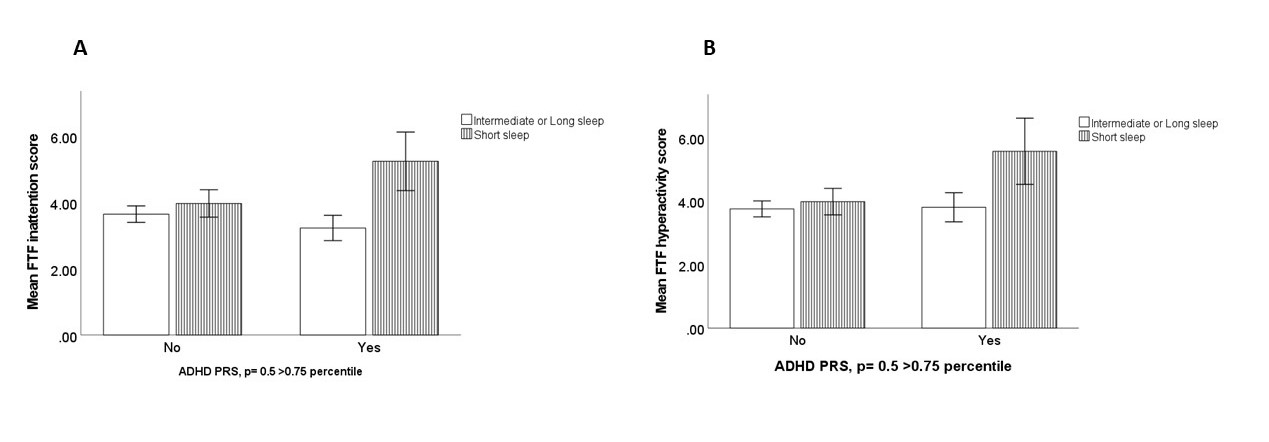


**Fig S1. ANOVA interactions between high PRS for ADHD, questionnaire-based short sleep across childhood and ADHD secondary outcomes (i.e. FTF-inattention and FTF-hyperacivity) at five years old.** Graph A displays the differences in relation to FTF-inattention score, and shows that those children with both questionnaire-based short sleep and high PRS for ADHD present the highest scores in FTF-inattention at five years old. Graph B displays the differences in relation to FTF-hyperactivity score, and shows that those children with both questionnaire-based short sleep and high PRS for ADHD present the highest scores in FTF-hyperactivity at five years old. The confidence interval represent +/-2 SE. X axes refers to the PRS for ADHD score (p-value threshold 0.50), which was dichotomized at the 75th percentile: ≥ 75th percentile (i.e. high=Yes) vs. lower (=No). Y axes refers to the total mean score for the outcome at five years old.
